# Effects of Propofol on Electrical Synaptic Strength in Coupling Reticular Thalamic GABAergic Parvalbumin-Expressing Neurons

**DOI:** 10.3389/fnins.2020.00364

**Published:** 2020-04-28

**Authors:** Yu Zhang, Chengxi Liu, Lin Zhang, Wenjing Zhou, Shouyang Yu, Rulan Yi, Dan Luo, Xiaoyun Fu

**Affiliations:** ^1^Department of Anesthesiology, Affiliated Hospital of Zunyi Medical University, Guizhou, China; ^2^Guizhou Key Laboratory of Anesthesia and Organ Protection, Zunyi Medical University, Guizhou, China; ^3^Guizhou Key Laboratory of Brain Science, Zunyi Medical University, Guizhou, China; ^4^Department of Critical Care Medicine, Affiliated Hospital of Zunyi Medical University, Guizhou, China

**Keywords:** electrical synapses, synaptic strength, thalamic reticular nucleus, propofol, general anesthesia

## Abstract

Electrical synapses between neurons exhibit a high degree of plasticity, which makes critical contributions to neuronal communication. The GABAergic parvalbumin-expressing (PV+) neurons in the thalamic reticular nucleus (TRN) interact with each other through electrical and chemical synapses. Plasticity of electrical synaptic transmission in TRN plays a key role in regulating thalamocortical and corticothalamic circuits and even the formation of consciousness. We here examined the effects of propofol, a commonly used general anesthetic agent, on the strength of electrical synapses between TRN PV+ neurons by fluorescence-guided patch-clamp recording and pharmacological methods. Results show that 100 μM propofol reduced the electrical synaptic strength between TRN PV+ neurons. Notably, the propofol-induced depression of electrical synaptic strength between TRN PV+ neurons was diminished by saclofen (10 μM, antagonist of GABA_B_ receptors), but not blocked by gabazine (10 μM, antagonist of GABA_A_ receptors). Application of baclofen (10 μM, agonist of GABA_B_ receptors), similar to propofol, also reduced the electrical synaptic strength between TRN PV+ neurons. Moreover, the propofol-induced depression of electrical synaptic strength between TRN PV+ neurons was abolished by 9-CPA (100 μM, specific adenylyl cyclase inhibitor), and by KT5720 (1 μM, selective inhibitor of PKA). Our findings indicate that propofol acts on metabotropic GABA_B_ receptors, resulting in a depression of electrical synaptic transmission of coupled TRN PV+ neurons, which is mediated by the adenylyl cyclase-cAMP-PKA signaling pathway. Our findings also imply that propofol may change the thalamocortical communication via inducing depression of electrical synaptic strength in the TRN.

## Introduction

Chemical and electrical synaptic communications are the most essential properties of a neural network, which are fundamental to the brain to receiving and integrating information from the environment (Smith and Pereda, 2003; Ovsepian and Vesselkin, 2014). However, unlike chemical synapses, electrical synapses have only recently been proved to show plasticity from milliseconds to days (Postma et al., 2011; Alcami and Pereda, 2019). This property enables electrical synapses to be constantly tuned to adapt to the ever-changing status of specific neural circuits. The effects of various general anesthetics on chemical synaptic strength have been studied by many past researches (Richards, 2002; Campagna et al., 2003; Vutskits, 2012), but the actions of general anesthetics on electrical synaptic strength, which may have played an important role in the mechanism of general anesthesia, have received little attention.

Gap junctions are the ultrastructural substrate of electrical synapses, which are small aqueous channels comprising connexins (Rash et al., 1998). Gap junctions work as a “bridge” between the membranes of neighboring neurons, which can quickly transmit intercellular current flow from one depolarized neuron to all the neighboring neurons. Hence electrical synapses are thought to synchronize the subthreshold and spike-mediated activity of these neurons (Sohl et al., 2005). Mechanisms that change membrane properties of the electrical coupling, or that alter the function of the connexins (such as changes in expression level or phosphorylation of the connexins) can change the strength of electrical synapses between two neighboring neurons (Del Corsso et al., 2012; Kothmann et al., 2012).

The power of electrical synapses to coordinate brain activity, such as “consciousness” of our concern in general anesthesia mechanism, should be critically dependent on the specific type and function of neurons that are electrically coupled. The thalamic reticular nucleus (TRN) modulates thalamocortical oscillations that result from the integrational activities of neural circuits between the cerebral cortex and thalamus (Fuentealba and Steriade, 2005). The TRN contains mainly parvalbumin-expressing (PV+) neurons, which use gamma-aminobutyric acid (GABA) as a transmitter and provide major inhibition for the thalamocortical neurons in the dorsal thalamus (Hou et al., 2016). GABAergic TRN neurons have been proven to provide critical contributions to the initiation of unconsciousness in both physiological and pathological states (Fuentealba and Steriade, 2005; Gottschalk and Miotke, 2009). In addition, our previous study found that changing noradrenergic inputs to TRN can modulate the unconsciousness time of general anesthesia (Zhang et al., 2019).

Thalamic reticular nucleus neurons communicate with each other primarily via electrical and chemical synapses (Landisman et al., 2002). So the electrical coupling of GABAergic neurons in TRN coordinated the excitatory input from corticothalamic and thalamocortical axons and the inhibitory output from TRN to the thalamic relay nuclei (Huntsman and Huguenard, 2006). Given the key role of TRN in the formation of consciousness, and the strength of electrical synapses of TRN GABAergic neurons, it is possible that general anesthetic act on specific receptors to selectively influence the strength of electrical synapses of TRN GABAergic neurons. Here, we evaluate the extent of electrical synaptic strength in coupled TRN PV+ neurons, and explore the effects of propofol (a short-acting intravenously administered anesthetic agent commonly used for general anesthesia) on the strength of electrical synapses.

## Materials and Methods

### Animals

All experiments detailed complied with the Guide for the Care and Use of Laboratory Animals (8th edition), and were authorized by the Zunyi Medical University Animal Care and Use Committees. Experiments were conducted with PV^tdTomato^ mice on a C57BL/6 background (Stock No: 028594), which were obtained from The Jackson Laboratory (SCR_004633). All mice were bred and raised in a pathogen-free animal holding facility with controlled room temperature (22–25°C) and 12-h light/dark cycle. Free access to standard chow diet and water were performed to all mice. Both male and female mice were used in this study.

Drugs administration. Propofol injectable emulsion (Diprivan, AstraZeneca) was diluted to 100 μM in artificial cerebrospinal fluid (ACSF) for perfusion. Gabazine (CAS:104104-50-9), saclofen (CAS:125464-42-8), baclofen (CAS:63701-55-3), and 9-Cyclopentyladenine monomethanesulfonate (9-CPA, CAS: 189639-09-6) were purchased from Sigma-Aldrich (SCR_008988). All drugs were dissolved in ACSF solution and applied to the recording slice at 1 ml/min. The application of antagonist, agonist or inhibitor was started 5 min before recording and lasting throughout each recording period.

Brain slice preparation. Mice with age 30–60 postnatal days were anesthetized with pentobarbital (1%, 50 mg/kg) and perfused transcardially with ice-cold oxygenated (95% O_2_ and 5% CO_2_) ACSF containing the following (in mM): 126 NaCl, 2.95 KCl, 26 NaHCO_3_, 1.25 NaH_2_PO_4_, 2 CaCl_2_, 10 D-glucose, and 2 MgCl_2_. The brains were removed after decapitation of mice, and fixed to the stage of a vibrating microtome (HM650V; Thermo Fisher Scientific). Sagittal thalamic slices containing the TRN were cut at a thickness of 250 μm in ice-cold ACSF and transferred to a holding chamber for incubation. The incubation included two periods, initially at 36°C for 30 min in oxygenated ACSF and then at room temperature for 30 min. In total 79 PV^tdTomato^ mice were used for brain slice preparation.

Electrical synaptic strength recording. Prepared TRN slices were transferred to a recording chamber perfused with oxygenated ACSF with a flow rate of 3 ml/min. TRN neurons were visually identified using an upright epifluorescence microscope (Olympus Optical) equipped with a Qimaging digital camera. TRN PV+ neurons were identified by somatic tdTomato fluorescence visualized by an LED illuminant (6500 K, Olympus). Recording pipettes were made of borosilicate glass using a P-97 puller (Sutter Instruments) and had resistances of 3.5–5.0 MΩ when filled with cesium-based intracellular solution to prevent the change of Rin and Vm, which containing the following (in mM): 120 CsOH, 120 gluconic acid, 4 NaCl, 2 MgCl_2_, 0.5 CaCl_2_, 5 HEPES-K, 0.2 EGTA, 2 ATP-Mg, 0.5 GTP-Na. The membrane potential was held constant at −70 mV to avoid changes in input resistance. Ten micrometer CNQX (Abcam) and 50 μM DL-AP5 (Abcam) were added to the ACSF to prevent the confounding effects of glutamatergic AMPA and NMDA transmission. The neuronal voltage signal was amplified by a 700B amplifier (Molecular Devices, Axon CNS), filtered at 1 kHz and digitized at 10 kHz.

For the electrical synaptic strength measurement and statistical analyses, we used repeat (1 s interval, 10 sweeps) hyperpolarizing current step (100 pA, 500 ms) to induce voltage responses of both cells. Voltage responses were recorded every 2 min during the whole recording period. The strength of electrical coupling is quantified by the coupling coefficient (cc) and coupling conductance (Gc), which are defined as the primary endpoint of this study. cc represents the ratio of the postsynaptic voltage deflection divided by presynaptic voltage deflection, but electrical transmissions are bidirectional, so we give inward current stimulation alternately to each neuron of a paired recording, and the cc is calculated as a mean value for each direction as follow: cc = (ΔV_1_/ΔV_2_ + ΔV_2_/ΔV_1_)/2. The calculation of the Gc is based on the transfer resistance (R_12_) as follow: G_C_ = 1/R_C_, where Rc = (R_1_R_2_−R_12_^2^)/R_12_, where R_1_ = ΔV_1_/I_inj_, R_2_ = ΔV_2_/I_inj_, and R_12_ = R_21_ = ΔV_1_/I_inj_ = ΔV_2_/I_inj_ (Bennett, 1966). Statistical analyses were performed with Clampfit10 (Axon CNS) and Prism8 (GraphPad). Data are presented as means ± SD. Changes in time-course of Gc were analyzed using a repeated-measures ANOVA on non-normalized data. Two-tailed paired *t*-test was used to determine whether different drug administration induced significant changes in electrical synaptic strength over a population of recorded data. A value of *p* < 0.05 was considered to be statistically significant.

## Results

### Electrical Coupling Characters of TRN Neurons

**FIGURE 1:**
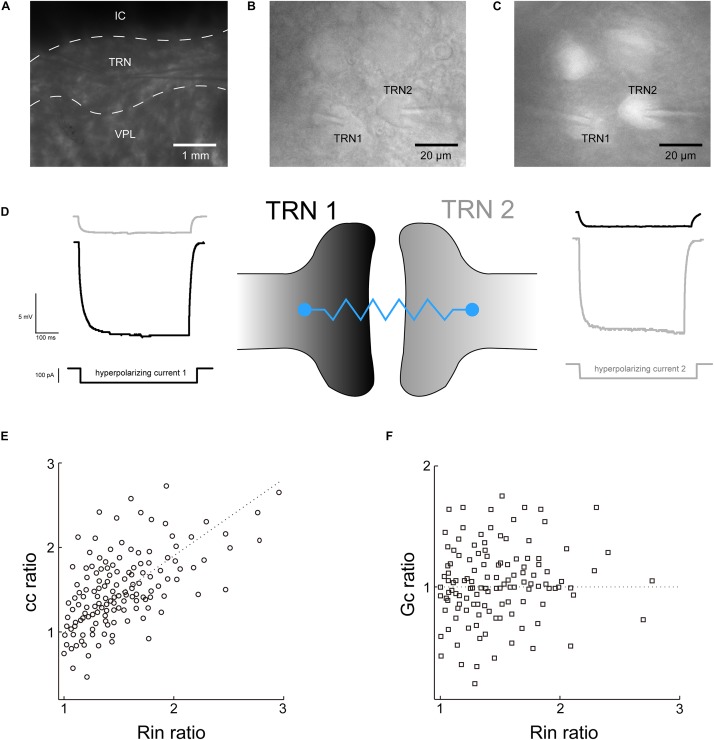
Recording of the electrical strength of coupled TRN neurons. **(A)** Low-magnification photomicrograph showing two recording electrodes in the TRN region of a thalamic slice preparation. IC, internal capsule; VPL, ventral posterolateral nucleus. **(B)** High-magnification view of a pair of TRN neurons (labeled TRN1 and TRN2) with adjoining somata with tips of recording electrodes. **(C)** The TRN neurons were confirmed as PV+ neurons by somatic tdTomato fluorescence. **(D)** Recording traces of voltage responses to hyperpolarizing current injected to each TRN neurons. Left, current injection into TRN 1 produced a direct voltage deflection of TRN 1 (black trace) and a non-direct (via electrical synaptic transmission) voltage deflection of TRN 2 (gray trace). Right is the reverse. **(E)** electrical transmission asymmetry of cc plotted against Rin ratio. **(F)** Electrical transmission asymmetry of Gc plotted against Rin ratio.

Recordings were made in the TRN nucleus (Figure 1A). Neighboring TRN PV+ neurons with space <30 μm apart were chosen to build paired recording (Figures 1B,C). Electrical transmissions were commonly recorded between pairs of TRN neurons, and the strength of electrical coupling is quantified by the cc and Gc. Both neurons were maintained at a baseline of −70 mV, and 100-pA current injection (500 ms) induced 10–30 mV voltage deflections in the presynaptic neuron (ΔV_1_), while resulting in a 1–1.5 mV voltage deflections in the postsynaptic neuron (ΔV_2_) (Figure 1D). Reported voltage deflections were induced by 10 repeated current steps and averaged. The averaged cc we recorded in TRN PV+ neurons was 0.075 ± 0.057 (*n* = 32 pairs). The coupling conductance Gc was calculated by using the current and voltage deflection in the coupled neurons as mentioned in the method section. Results show that Gc in TRN PV+ neurons was 0.42 ± 0.15 nS (*n* = 30 pairs). We then plotted the cc (cc2/cc1) and Gc (Gc2/Gc1) ratio against the input resistance (Rin) ratio (R2/R1) for a cohort to explore whether neurons with larger Rin in paired neurons would determine the direction of electrical transmission asymmetry. Results show that cc asymmetry generally increases with Rin ratio (Figure 1E), indicating that the direction of cc asymmetry could be affected by Rin ratio. While Gc was not subject to the influence of the Rin ratio (Figure 1F), thus Gc was chosen as the measurement of the electrical coupling strength of TRN PV+ neurons in the next step of the experiment.

### Propofol Reduces the Electrical Synaptic Strength Between TRN PV+ Neurons

**FIGURE 2:**
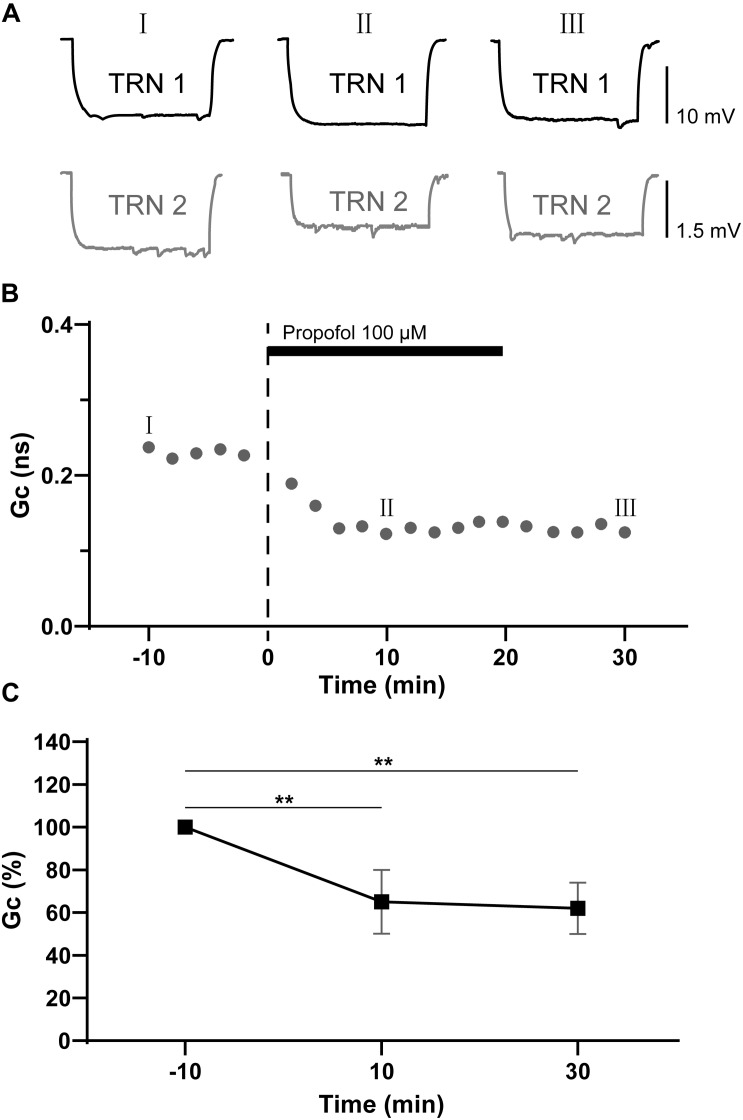
Propofol reduces the electrical synaptic strength of electrical coupled TRN neurons. **(A)** Example traces of voltage responses of a coupled pair of TRN neurons before (I), during (II), and after (III) propofol application (100 μM). **(B)** Time course calculations of Gc for traces recorded from the pair of TRN neurons shown in **A**, correspondence time points of the traces in **A** were marked as I to III. **(C)** Average Gc values taken 10 min before, 10 min after propofol application, and 10 min after propofol washout (means ± SD, *n* = 21 pairs). Average Gc values at 10 min before propofol application and 10 min after drug washout were taken to calculate ΔGc. **p < 0.01, repeated-measures ANOVA.

We next added propofol (100 μM) into the perfusate to test its effect on the electrical coupling strength of TRN PV+ neurons. Study in humans indicate the concentration of propofol in CSF during surgical anesthesia is above 70 μM, which is in agreement with its high lipophilicity (Ludbrook et al., 2002). In a rat, the concentration of propofol in the brain was found to be 88 μM (Shyr et al., 1995). So, the 100 μM concentration used in our experiments are therefore chosen based on the clinically effective ranges of propofol. The Rin of TRN PV+ neurons did not show any obvious change before versus after propofol application. The postsynaptic voltage responses of the coupled TRN neurons decreased significantly following propofol application, and the depression lasts for 10–30 min after propofol washout (Figures 2A,B). Tests were repeated in 10 pairs of electrical coupling TRN neurons, the coupling strength depressed significantly with ΔGc = −35 ± 5% (*p* = 0.003, two-tailed paired *t*-test; Figure 2C). These results suggest that propofol significantly reduced the electrical synaptic strength between coupled TRN PV+ neurons.

### Blockade of GABA_A_ Receptors Failed to Change the Propofol-Induced Depression of Electrical Synaptic Strength

**FIGURE 3:**
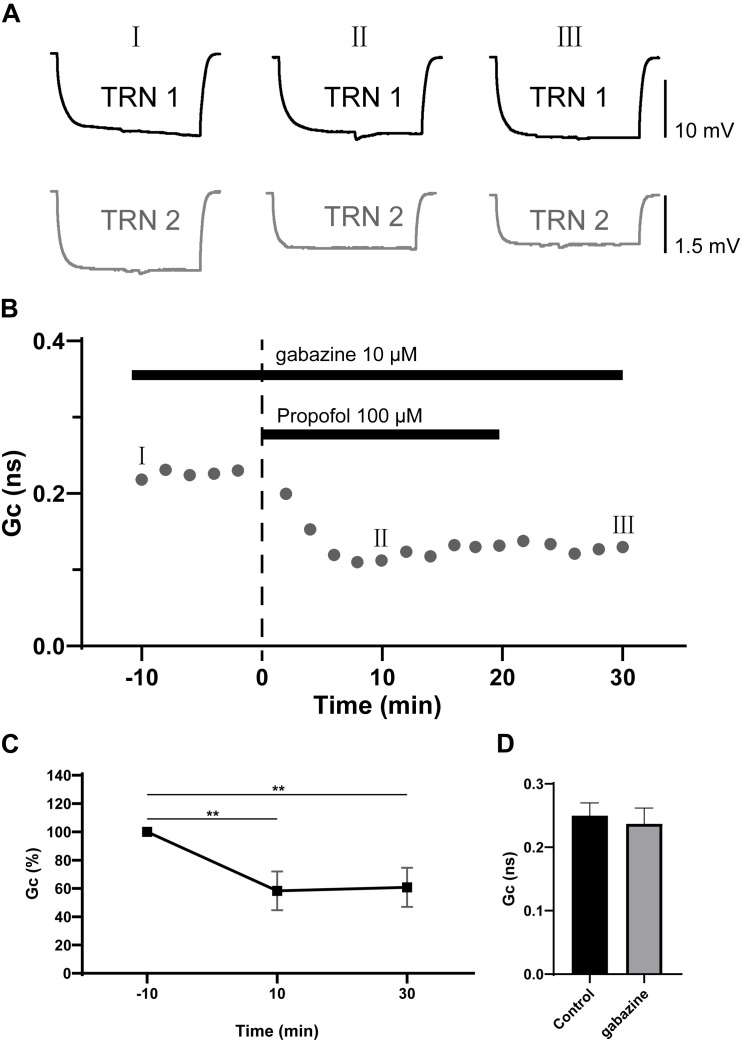
Effects of GABA_A_ receptor antagonist gabazine on propofol-induced depression of electrical synaptic strength of TRN neurons. **(A)** With 10 μM gabazine in the ACSF perfusion, example traces of voltage responses of a coupled pair of TRN neurons before (I), during (II), and after (III) propofol application. **(B)** Time course calculations of Gc for all traces of the pair of TRN neurons shown in **A**, correspondence time points of the traces in **A** were marked as I to III. **(C)** Average Gc values taken 10 min before, 10 min after propofol application, and 10 min after propofol washout (means ± SD, *n* = 15 pairs). **(D)** Compare of the mean and deviation of Gc (in 10 min) in control conditions, and in 10 μM gabazine. Average Gc values at 10 min before propofol application and 10 min after drug washout were taken to calculate ΔGc. **p < 0.01, repeated-measures ANOVA.

Propofol has been proposed to potentiate GABA_A_ receptor, and even activate GABA_A_ receptors directly at high doses (Concas et al., 1992). We further examined whether the propofol-induced depression of electrical synaptic strength of TRN PV+ neurons was caused by GABA_A_ receptor activation by repeating the experiment in the presence of the GABA_A_ receptor antagonist gabazine (10 μM). As shown in Figure 3, with gabazine (10 μM) application, propofol 100 μM still induced a significant depression of the coupling strength as ΔGc = −37 ± 6% (*p* = 0.012, two-tailed paired *t*-test; Figures 3B,C). When compared to control (pre-propofol), 10 μM gabazine did not change Gc (Figure 3D). These results suggest that GABA_A_ receptors were not involved in propofol-induced depression of electrical synaptic strength of TRN PV+ neurons.

### The Propofol-Induced Depression of Electrical Synaptic Strength of TRN PV+ Neurons Was Mediated by GABA_B_ Receptor

**FIGURE 4:**
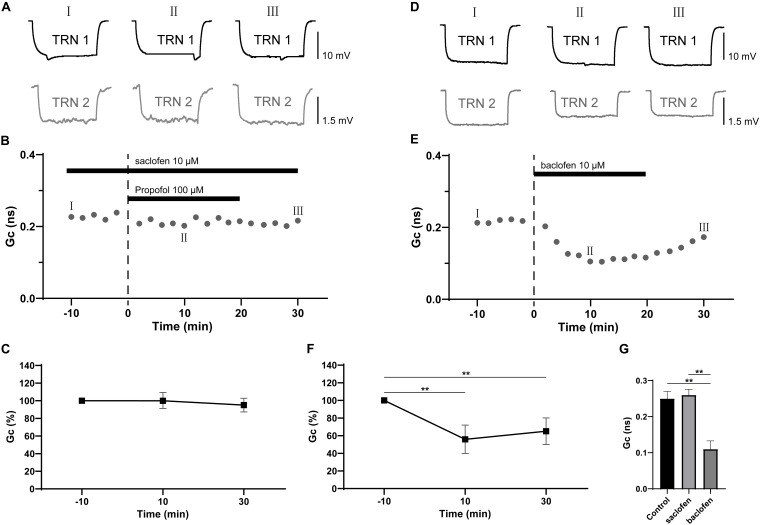
Effects of GABA_B_ receptor antagonist saclofen on propofol-induced depression of electrical synaptic strength of TRN neurons. **(A)** With 10 μM saclofen in the ACSF perfusion, example traces of voltage responses of a coupled pair of TRN neurons before (I), during (II), and after (III) propofol application. **(B)** Time course calculations of Gc for all traces of the pair of TRN neurons shown in **A**, correspondence time points of the traces in **A** were marked as I to III. **(C)** Average Gc values taken 10 min before, 10 min after propofol application, and 10 min after propofol washout (means ± SD, *n* = 15 pairs). **(D)** example traces of voltage responses of a coupled pair of TRN neurons before (I), during (II), and after (III) baclofen (10 μM) application. **(E)** Time course calculations of Gc for all traces of the pair of TRN neurons shown in **D**, correspondence time points of the traces in **D** were marked as I to III. **(F)** Average Gc values taken 10 min before, 10 min after propofol application, and 10 min after propofol washout (means ± SD, *n* = 10 pairs). **(G)**, compare of the mean and deviation of Gc (in 10 min) in control conditions, and in 10 μM saclofen and baclofen. Average Gc values at 10 min before drug application and 10 min after drug washout were taken to calculate ΔGc. **p < 0.01, repeated-measures ANOVA.

GABA_B_ receptors are metabotropic transmembrane receptors coupled to a number of cellular effectors mechanisms underlying neuronal signal modulation (Terunuma, 2018). It has been demonstrated that GABA_B_ receptors partially contributed to propofol-induced depression of neuron activity (Schwieler et al., 2003). We hypothesized that GABA_B_ receptors may take part in propofol-induced depression of electrical synaptic strength of TRN PV+ neurons. We next applied the GABA_B_ receptor selective antagonist, saclofen (10 μM) (Xuan et al., 2018) to test its effect on propofol-induced inhibition of electrical coupling strength of TRN PV+ neurons. As shown in Figures 4A–C, in the presence of saclofen, propofol failed to reduce the electrical synaptic strength, indicating the propofol-induced depression of electrical synaptic strength of TRN PV+ neurons were mediated by GABA_B_ receptors. Further, we examined whether pharmacological activation of GABA_B_ receptor will change the electrical synaptic strength of TRN PV+ neurons by perfusing ACSF containing the GABA_B_ receptor selective agonist, baclofen (10 μM). As shown in Figures 4D–F, baclofen transiently reduces the coupling strength as: ΔGc = −45 ± 7% (*p* = 0.015, two-tailed paired *t*-test; Figures 4B,C), but the inhibition of Gc started to restore immediately after the end of the baclofen administration (Figure 4E), which was different from the depression of electrical synaptic strength induced by propofol. When compared to control (pre-propofol), 10 μM saclofen did not change Gc (Figure 4G).

### Propofol-Induced Depression of Electrical Synaptic Strength of TRN PV+ Neurons Mediated by the Adenylyl Cyclase Signaling Cascade

**FIGURE 5:**
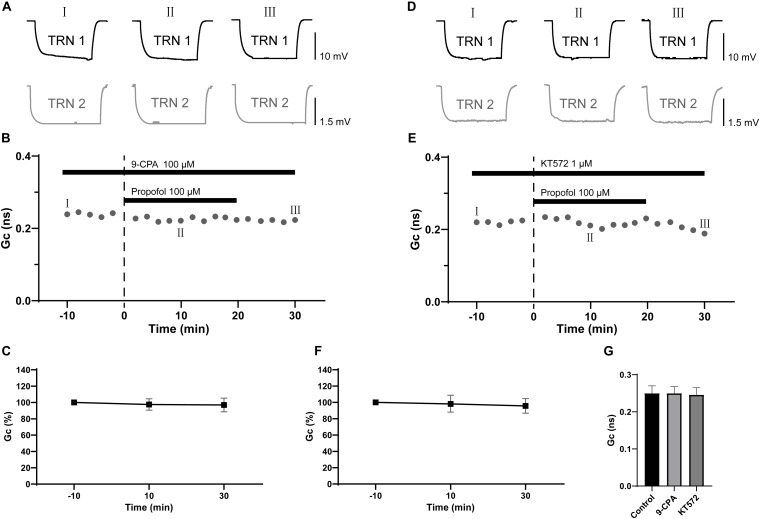
Effects of specific inhibitor of adenylyl cyclase and PKA on propofol-induced depression of electrical synaptic strength of TRN neurons. **(A)** With 100 μM 9-CPA in the ACSF perfusion, example traces of voltage responses of a coupled pair of TRN neurons before (I), during (II), and after (III) propofol application. **(B)** Time course calculations of Gc for all traces of the pair of TRN neurons shown in **A**, correspondence time points of the traces in **A** were marked as I to III. **(C)** Average Gc values taken 10 min before, 10 min after propofol application, and 10 min after propofol washout (means ± SD, *n* = 7 pairs). **(D)** With 1 μM KT5720 in the ACSF perfusion, example traces of voltage responses of a coupled pair of TRN neurons before (I), during (II), and after (III) propofol application. **(E)** Time course calculations of Gc for all traces of the pair of TRN neurons shown in **D**, correspondence time points of the traces in **D** were marked as I to III. **(F)** Average Gc values taken taken 10 min before, 10 min after propofol application, and 10 min after propofol washout (means ± SD, *n* = 9 pairs). **(G)** Compare of the mean and deviation of Gc (in 10 min) in control conditions, and in 100 μM 9-CPA and 1 μM KT572. Average Gc values at 10 min before propofol application and 10 min after drug washout were taken to calculate ΔGc.

It has been reported that activation of GABA_B_ receptors leads to a potentiation of the activity of adenylyl cyclase (Olianas and Onali, 1999), while adenylyl cyclase is established to decrease the electrical coupling between hippocampal interneurons (Zsiros and Maccaferri, 2008). Therefore, we tested whether adenylyl cyclase was necessary for the propofol-induced depression of electrical synaptic strength of TRN PV+ neurons. In the presence of a specific adenylyl cyclase inhibitor, 9-CPA (100 μM), there was no significant change in the electrical coupling strength following application of propofol (Figures 5A–C), which suggests that activation of adenylyl cyclase is crucial in propofol-induced depression of electrical synaptic strength of TRN PV+ neurons. Activation of adenylyl cyclase is known to increase the intracellular levels of cAMP, which in turn causes an elevation of PKA (Bauman et al., 2006; Yan et al., 2011; Chan and Lutfy, 2016). So, we further examined whether inhibition of PKA mediates propofol-induced depression of electrical synaptic strength. In the presence of KT5720 (1 μM), a selective PKA inhibitor, propofol failed to reduce the electrical synaptic strength of TRN PV+ neurons (Figures 5D–F). When compared to control (pre-propofol), 100 μM 9-CPA and 1 μM KT5720 did not change Gc (Figure 5G). Together, these results suggest that adenylyl cyclase-cAMP-PKA signaling cascade regulates propofol-induced depression of electrical synapses in TRN PV+ neurons.

## Discussion

The present study showed that propofol decreased the electrical synaptic strength of electrical coupled TRN PV+ neurons. The depression of electrical synaptic strength induced by propofol was diminished by the administration of antagonist of GABA_B_ receptors and inhibitor of adenylyl cyclase. Our results indicate that propofol acts on metabotropic GABA_B_ receptors, leading to a potentiation of adenylyl cyclase, which results in a depression of electrical synaptic transmission of coupled TRN PV+ neurons. Generally speaking, the electrical synaptic transmission of coupled TRN PV+ neurons can be modulated by the activation of metabotropic GABA_B_ receptors. Our findings are supported by a prior study that showed the strength of electrical synapses of TRN neurons can be modulated by GABAergic input (Lefler et al., 2014).

### Signaling Cascades Involved in the Propofol-Induced Depression of Electrical Synapses in the TRN PV+ Neurons

GABA_B_ receptors are G protein-coupled receptors that are involved in both presynaptic and slow postsynaptic inhibition (Pin et al., 2009; Rondard et al., 2011). Propofol has been proved to depresses the presynaptic release of glutamate by activating presynaptic GABA_B_ receptors (Xuan et al., 2018). In this study, our results focused on another pathway of GABA_B_ receptors in modulation of neuronal activities: from GABA_B_ receptors to adenylyl cyclase, an enzyme that converts ATP to the most important second messenger – cAMP (Dessauer et al., 2017). Our results showed that a specific inhibitor of adenylyl cyclase and PKA diminished the depression of electrical synaptic strength induced by propofol, indicating that adenylyl cyclase-cAMP-PKA signaling pathway regulates the propofol-induced depression of electrical synapses in TRN PV+ neurons. But the actions of GABA_B_ receptors on adenylyl cyclase are controversially dependent upon the specific type of subunit of G protein that binds to GABA_B_ receptors. For example, Gαi G-proteins inhibits adenylyl cyclase, reducing cAMP levels and downstream kinase activity (Federman et al., 1992). But Gβγ proteins were found to increase the activity of adenylyl cyclase (Robichon et al., 2004). Taken together, by testing the effects of antagonist of GABA_B_ receptors and inhibitors of adenylyl cyclase-cAMP-PKA signaling pathway, we observed that propofol-induced depression of electrical synaptic strength in TRN PV+ neurons were mediated by the GABA_B_ – adenylyl cyclase – cAMP – PKA signaling cascade. But whether the effect of propofol is due to the increase or decrease of cAMP and PKA activity is still needed to be demonstrated by further study. Otherwise, the baclofen induced suppression shows partial recovery during the 10-min washout (Figure 4), but propofol 100 μM showed no recovery during post 20 min, which indicated that the concentration of propofol may produce a persistent activation of the GABAB receptor.

### The Potential Effects of Propofol on the Modulation of Electrical Synaptic Strength in TRN

Loss of consciousness is the most obvious efficacy of propofol as a general anesthetic. At loss of consciousness concentrations, propofol induces specific changes in electroencephalogram (EEG) rhythms of mammalian brain: “spindles,” which was characterized by EEG oscillation of 12–14 Hz, lasting several seconds (Ferenets et al., 2006). Our earlier study showed that spindles (8–12 Hz) can also be detected in rats under propofol anesthesia (Zhang et al., 2014). So the appearance of spindles is well correlated with the propofol-induced loss of consciousness.

The TRN is considered to play a central role in regulating the thalamocortical interactions (Fuentealba and Steriade, 2005). Many studies showed that inhibitory GABAergic TRN neurons are the key generator of sleep spindles, which is a type of thalamocortical EEG oscillation during sleep (Halassa et al., 2011; Fan et al., 2017a, b). In the TRN, most compact clusters of neurons are interconnected by electrical synapses, which synchronize the activity of these electrical-bonded neurons (Landisman et al., 2002). The electrical synapses of TRN neurons show relatively strong low-pass filter properties that lead to the synchronization of low-frequency events (Lee et al., 2014).

Therefore, modulation of electrical synaptic strength in the TRN potentially modifies the information transmission within the thalamocortical system. When the sensory input is blocked during general anesthesia, the propofol-induced depression of electrical synaptic strength in the TRN may reduce the dimensionality of the thalamocortical network, induce disconnection of larger cortical regions (Ching et al., 2010), make them insensitive to exogenous input, thus leads directly to the loss of consciousness. However, more experiments still need to prove the link between the depression of electrical synaptic strength in the TRN to hypersynchrony of larger cortical regions, or even the increased spindle activity.

## Conclusion

Taken together, our present study showed that propofol inhibited the electrical synaptic strength in the TRN PV+ neurons. Propofol reduces the electrical synaptic strength by facilitating GABA_B_ receptors and downstream adenylyl cyclase-cAMP signaling pathway.

## Data Availability Statement

All datasets generated for this study are included in the article.

## Ethics Statement

This study was carried out in accordance with the principles of the Basel Declaration and recommendations of the Guide for the Care and Use of Laboratory Animals, Zunyi Medical University Animal Care and Use Committees. The protocol was approved by the Zunyi Medical University Animal Care and Use Committees.

## Author Contributions

YZ and XF contributed the conception and design of the study. YZ and LZ organized the database. CL performed the statistical analysis. YZ wrote the first draft of the manuscript. WZ, RY, DL, and SY wrote sections of the manuscript. All authors contributed to manuscript revision, read and approved the submitted version.

## Conflict of Interest

The authors declare that the research was conducted in the absence of any commercial or financial relationships that could be construed as a potential conflict of interest.
